# Differentiating Pigs from Wild Boars Based on *NR6A1* and *MC1R* Gene Polymorphisms

**DOI:** 10.3390/ani11072123

**Published:** 2021-07-17

**Authors:** Anna Koseniuk, Grzegorz Smołucha, Małgorzata Natonek-Wiśniewska, Anna Radko, Dominika Rubiś

**Affiliations:** National Research Institute of Animal Production, Department of Animal Molecular Biology, Krakowska Street 1, 32-083 Balice, Poland; grzegorz.smolucha@iz.edu.pl (G.S.); malgorzata.natonek@iz.edu.pl (M.N.-W.); anna.radko@iz.edu.pl (A.R.); dominika.rubis@iz.edu.pl (D.R.)

**Keywords:** pig, wild boar, *MC1R*, *NR6A1*, genomes admixture, population structure

## Abstract

**Simple Summary:**

Wild boar meat is much more expensive than pork. Therefore, there are cases when pork is added to wild boar meat products, but this information is not included on the product label. Currently, there is no fully reliable method that would allow the identification of wild boar and domestic swine products. In this study, we tested the possibility of distinguishing two subspecies using polymorphisms within the *MC1R* and *NR6A1* genes. For this purpose, we used two techniques commonly used in molecular biology, PCR-RFLP and Real-time PCR.

**Abstract:**

This preliminary study aimed to differentiate domestic pigs from wild boars based on *MC1R* and *NR6A1* polymorphisms and to identify admixture between these genomes. We studied samples obtained from wild boars from two regions of Poland and five pig breeds: Polish Landrace, Polish Large White, Złotnicka White, Pulawska and Duroc. Along the *MC1R* gene sequence, we identified four polymorphic loci comprising three codons. The “wild type” allele was primarily found in wild boar but also in the Duroc and Złotnicka White breeds. Non-wild type alleles were identified in the vast majority of domestic pig samples and in two wild boar samples. Based on *MC1R* profiles, we conducted a population study, and revealed admixture between both genomes using STRUCTURE and NETWORK Software. Interestingly, an allelic discrimination assay with *NR6A1* g.748C > T TaqMan probes revealed a clear separation of samples into two groups: wild boar samples representing the C allele and domestic breeds representing the T allele. Based on the obtained results, we conclude that *NR6A1* g.748C > T is an effective marker for differentiating between wild boars and domestic pigs, where this is supported by *MC1R* data, to identify admixed profiles. We recommend that a larger sample of genomes is studied to verify this method.

## 1. Introduction

Conscious consumers search for food products consisting of defined reliable components. Wild boar meat products appear to be healthier than farmed pork products because the animals grow in a natural environment without any artificial nutritional additives. Although a porcine component can be simply identified, the differentiation between wild boar and domestic pig in meat products is still challenging [1,2]. The melanocortin 1 receptor (*MC1R*) gene, also called the extension locus (E), plays a key role in the pigmentation of skin and hair in mammals by regulating the synthesis of two dyes: eumelanin (brown to black) and pheomelanin (cream to red) in melanocytes [3]. Binding of *MC1R* to the α-melanocyte-stimulating hormone (αMSH) stimulates eumelanin production. The opposite effect results when ASIP (Agouti Signaling Protein) binds to αMSH, which causes the synthesis of pheomelanins. These genes show epistatic interaction [4,5]. In studies conducted by [6,7] on domestic pigs (*Sus scrofa f. domestica*) and Central European wild boars (*Sus scrofa scrofa*), several *MC1R* alleles were identified, one of which (E+) is considered the “wild” allele, and has been predominantly assigned to the wild boar population. Other alleles are prevalent in domestic pig breeds: dominant black (ED1 and ED2), black spotting (EP), and red (e) [6,7]. 

In [8], the authors conducted the first study aiming to differentiate between wild boars and commercial pig breeds based on combining both nuclear (melanocortin 1 receptor, *MC1R*) and mitochondrial DNA (D-loop). This analysis of *MC1R* proved to be more effective than the most common method used for species identification based on polymorphisms of mitochondrial DNA. The subspecies also differ in vertebrae; wild boars usually have 19 thoracic and dorsal vertebrae, whereas domestic pig breeds have 21–23. In [9], the authors identified a missense substitution (p.Pro192Leu) in the nuclear receptor subfamily 6, group A, member 1 (*NR6A1*) gene as a causative mutation of a QTL, which affects the number of vertebrae in pigs. 

In genetic research, the analysis of the history of breed development is of great importance. After World War II started, Poland begun breeding work aimed to stabilise pure breeds of pigs, sheep, and horses and keep primitive native breeds from extinction [10]. The Puławska breed is the first native breed of pigs in Poland. It was created by crossing the regional primitive breeds with Berkshire and English Large White breeds. The pigs are spotted, black and white, with a prevailing black colour [11]. The tri-colour is also accepted: black-white, red and black, with white spots in the lower part of the snout, legs and tail (accessed on 25 May 2021; http://www.bioroznorodnosc.izoo.krakow.pl/swinie/pulawska; in Polish). The Polish landrace was settled using German and Swedish national breeds. The landrace pigs are white-skinned and covered with white hair. Small dark spots are also acceptable (accessed on 25 May 2021; https://www.polsus.pl/index.php/en/pig-breeding/breeds/polish-landrace). The Polish Large White breed was created before World War II, although unfortunately, these breeding achievements were lost. The current breed was settled in 1947 based on large white pigs imported from England and Sweden. Polish Large White (PLW) individuals are characterized with white/pale hair and skin (accessed on 25 May 2021; https://www.polsus.pl/index.php/en/pig-breeding/breeds/polish-large-white). The Złotnicka White breed has a similar history of development to the PLW breed, creating a new breed being undermined after World War II. The current breed was created in the 1950s based on local primitive long-eared and short-eared pig breeds [10]. Duroc was imported to Poland in the 1970s to improve the meat performance of Polish breeds [12]. Its coat is in shades of red, from light gold to mahogany [6]. There was variation in coat colour among wild boar individuals and within each other breed included in the study (Figure S2).

This study aimed to verify whether specific polymorphisms of *MC1R* and *NR6A1* sequences are possible markers for differentiating products made from Polish domestic pigs (*Sus scrofa domestica*) or Central European wild boars (*Sus scrofa scrofa*) and identifying admixture among populations.

## 2. Materials and Methods

### 2.1. Material

#### Sampling

DNA isolation was performed using the Sherlock AX reagent kit (company, A&A Biotechnology, Gdynia, Poland), according to the protocols provided by the manufacturer. Sample DNA concentration and purity were quantified using a Nanodrop 2000 spectrophotometer (Thermo Fisher Scientific, Waltham, MA, USA). The purity of the DNA samples were estimated on the basis of the absorbance ratio of 260/280 and in all samples were at the level of 1.8, which proves the high purity of the obtained DNA. All DNA samples were normalized to 100 ng/µL. The DNA was extracted from the ear tissues of 22 wild boars from two Polish regions and blood or muscle tissues from 66 pigs comprising 5 breeds: Polish Landrace (PL, *n* = 14; 2 farms), Polish Large White (PLW, *n* = 13; 2 farms), Złotnicka White (ZW, *n* = 13; 1 farm), Pulawska (P, *n* = 10; 2 farms) and Duroc (D, *n* = 17; 2 farms) (Figure S1). To avoid using related samples, parentage was established for all samples based on 14 microsatellites recommended by the International Society of Animal Genetics. We also studied 5 samples representing hybrids of both subspecies, which were constituted from randomly selected DNA samples representing both wild boar and domestic pig.

Wild boars were hunted in 2009–2011 during hunting seasons. Licensed hunters collected samples as part of routine wildlife management. Blood samples from pigs were delivered by representatives of the Polish Pig Breeders’ Association (POLSUS) as part of a routine parentage control conducted at the National Research Institute of Animal Production, Balice, Poland, therefore no local ethics commission permission was needed.

### 2.2. Methods

#### 2.2.1. PCR Amplification, RFLP Genotyping and Sequencing of *MC1R*

All samples were sequenced and RFLP genotyped according to the following methodology. Amplification of a 575 bp *MC1R* gene fragment was performed in a total volume of 25 μL, comprising 2 μL 30–50 ng genomic DNA, 1U HotStarTaq DNA Polymerase (Qiagen, No. 203205, Germany), 1× PCR buffer, 0.3 mM MgCl2, 1× Q-Solution, 0.4 μM dNTP (10 mM; Thermo Fisher Scientific, Waltham, MA, USA, ) and forward and reverse primers (0.2 μM each) (Table 1 and Table S1).

For a quick discriminating test, we used a RFLP method. The PCR products were digested in a total volume of 20 μL, consisting of 10 μL PCR products, 1× reaction buffer, 1 U of each restriction enzyme (BspHI, BstUI; Thermo Fisher Scientific, Waltham, MA, USA city, state abbr). Incubation time for the BstUI enzyme was 1 h at 60 °C and for BspHI was 14 h at 37 °C. The digestion was conducted separately for each enzyme. Digested products of *MC1R* were separated on 2% agarose gel by electrophoresis. 

Direct sequencing of *MC1R* was conducted for all samples. The sequencing protocol was conducted using a BigDye v.3.1 reagents set according to the manufacturer’s protocol (Thermo Scientific, Waltham, MA, USA), using the PCR primers specified in Table 1 and Table S1. Capillary electrophoresis was performed in an automatic sequencer 3500xl (Applied Biosystems, Foster City, CA, USA). The resulting sequences, together with sequences of *MC1R* alleles deposited in GeneBank (Accession numbers specified in Table 2) were aligned using a ClustalW alignment implemented in the BioEdit sequence editor [13].

#### 2.2.2. Phylogenetic and Admixture Analysis

To analyse the population structure and patterns of admixture among pig and wild boar populations, we aligned the samples studied with specific sequences of previously identified *MC1R* alleles (Table 2), and analysed these using Structure v.2.3.4 software [14]. The following settings were used: admixture model, correlated allele frequencies among populations, and no population information (run 10 times for each K, initially from K1 to K10). The following parameter settings were applied: 10 iterations with a 100,000 burn-in period and 200,000 MCMC reps. We used Clumpak v1.1 software to visualize the results. The optimal K for this analysis was inferred using Structure Harvester [15] software based on a method proposed by [16]. We used DnaSP v.6.0 [17] to generate haplotype files of domestic pig and wild boar sequences. To study the genetic differentiation of domestic and wild boar populations, we computed a Fst [18] based on haplotype files using GENEPOP v.4.7.5 [19,20]. Finally, we generated a haplotype network using NETWORK 4.6.1.6 [21].

#### 2.2.3. *NR6A1* Gene Study and Real Time PCR

Before Sanger sequencing, PCR reaction with the *NR6A1* target primers was performed in a total volume of 15 μL. Specifically, we used: 1 μL DNA (30–50 ng/μL), HotStarTaq DNA Polymerase (Qiagen No.203205,Hilden, Germany) containing 1 U Polymerase, 1× PCR Buffer, 1× Q-Solution, 0.3 mM MgCl_2_, and forward and reverse primer (0.4 mMol each). Specific primers are depicted in Table 1, and thermal conditions in Table S1. The PCR product was purified using EXOSAP-it enzyme (Affymetrix, Santa Clara, CA, USA) and sequenced from both complementary strands. Sanger sequencing was carried out using reagent BigDye v.1.1 for short sequence analysis (Thermo Fisher Scientific, Waltham, MA, USA). The electrophoresis was performed in a 3500xL Genetic analyser (Applied Biosystems, Foster City, CA, USA city, state abbr). Two randomly selected samples were sequenced, one from the pig samples and one from the wild boar samples. To confirm that *NR6A1* sequences were obtained, these sequences were compared to the NCBI database using BLAST.

An Allelic Discrimination Assay (ADA) with the use of TaqMan probes (Table 1) was conducted to determine a C > T allele at locus g.748 *NR6A1* (Accesion No AB248749). ADA was performed using all samples representing wild boars, pigs and five hybrids, conducted in StepOne Plus instrument (Applied Biosystems, Foster City, CA, USA city, state abbr). Two reaction controls were included in each amplification, i.e., one positive control (containing pig DNA at 30 ng/µL) and one negative control (reagents without DNA). The thermal conditions for real-time PCR are summarized in Table S1.

## 3. Results

### 3.1. MC1R 

#### 3.1.1. Sequence Study

A 575 bp long *MC1R* gene sequence was obtained (beginning at position 181 of the *MC1R* reference sequence No. NM_001008690 GeneBank). Sequence study of the analysed breeds and the reference sequence allowed us to identify four polymorphic sites (Table 1): c.370G > A (p.Asp124Asn), c.491C > T (p.Ala164Val), c.727G > A and c.729A > G (p.243Ala > Ala/Thr). Since the samples studied were c.729 GG monomorphic, the SNP c.729A > G was excluded from further analysis (Table 2).

The A (c.370A) allele was found in the homozygous (AA) form in samples representing the PL, PLW and P breeds. In the ZW breed, we identified eleven AA homozygotes and two heterozygotes (AG). The allele c.370G was found in the homozygous form (GG) in all but two wild boars, which were heterozygotes (AG). In the Duroc breed, 15 samples were GG homozygotes, one individual was heterozygous (AG) and one was homozygous for the A allele (AA). 

The c.491C > T mutation was mostly present in the Duroc breed (14 samples were TT homozygotes, one was a heterozygote and remaining two samples were CC homozygotes). However, the c.491 T allele was also found for the first time in the ZW breed. The remaining samples of ZW and other pig breeds (PL, PLW and P) and wild boars were all c.491 CC homozygotes. 

The polymorphism c.727G > A was identified in 15 Duroc pigs, where 14 samples were AA homozygotes, and one sample was an AG heterozygote. Two of the Duroc samples were c.727GG. In the ZW breed, two AG heterozygotes were identified.

#### 3.1.2. RFLP Genotyping

The non-synonymous G > A mutation at c.370 (p.Asp124Asn) was identified using the BspHI enzymatic assay (Table 1). Analysis of the electrophoretic separation of the PCR-RFLP reaction revealed the presence of two bands with lengths of 472 bp and 200 bp in AA homozygotes; the presence of three bands characterized AG heterozygotes: 672 bp, 472 bp and 200 bp; one band with a length of 672 bp (lack of enzymatic digestion) was observed for the GG homozygotes. 

Gel electrophoresis of the products of the BstUI enzyme digestion of c.727GG homozygote samples showed three visible bands (289 bp, 222 bp and 110 bp), which were identified in samples of PL, PLW, Pulawska breed. Most of the Duroc breed samples were characterised by a two band pattern (332 bp and 289 bp) due to the c727G >A mutation preventing enzymatic digestion at this locus. One Duroc c727AG heterozygote sample was characterised by four bands (332 bp, 289 bp, 222 bp and 110 bp), and one Duroc c.727GG homozygote by three bands (289 bp, 222 bp and 110 bp).

#### 3.1.3. Phylogenetic and Population Characteristics of Wild Boars and Domestic Pigs

**Figure 1:**
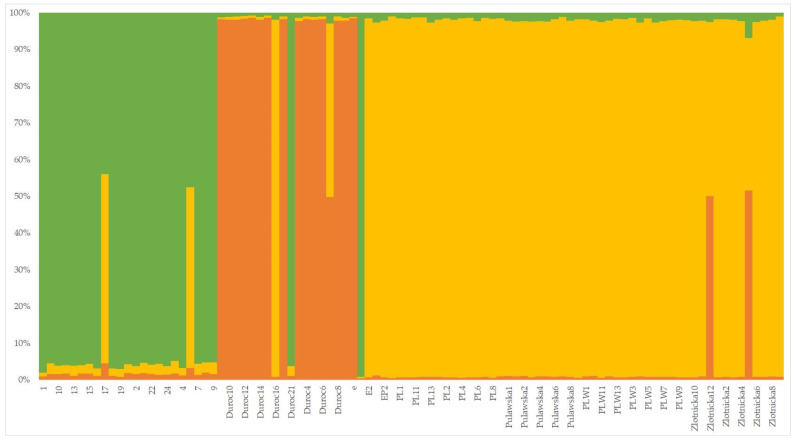
A pattern of *MC1R* admixed profiles in analysed samples; the computed number of expected population is K = 3; allele sequences are available on GeneBank: E^+^ (AF082490, [6]), E^D1^ (AF082489, [6]); e (EU443691, [7]), E^P2^ (EU443722, [7]), E^D2^ (EU443685, [7]), E^P3^ (EU443726, [7]).

In order to reveal the genetic structure of both subspecies, STRUCTURE software was used. The analysis revealed subdivision of the studied animals into three subpopulations (optimum K = 3, according to [16]) corresponding to the domestic pig, Duroc and wild boar populations. We also identified phenotypically wild boar individuals, which presented with a hybrid domestic and wild genotype (Figure 1). Two wild boar sample profiles genotypically demonstrated admixture from the domestic pig genome. Both samples have been identified to be *MC1R* c.370 A/G heterozygotes. In addition, one Duroc sample genetically clustered with the wild boar population. Samples were assigned to four haplotypes (H1–H4; Supplemantary file 1). Haplotype network revealed that H1 (predominant Duroc samples) is most distant from H2 (wild boar samples) in the mean of nt changes, while the number of different nt between H3 and H4 is comparable between H1 and H3 (Figure S3). Fst computed between two population (domestic pig and wild boar) was 0.3573.

### 3.2. NR6A1 Allele Discrimination

The default baseline settings were used to calculate a threshold of 0.47 for the T allele and 0.28 for the C allele. The threshold cycles (Ct) were set from 18 to 22 cycles and 20 to 28 cycles. The *NR6A1* allele discrimination protocol revealed the presence of the CC genotype in all samples designated as wild boar and the TT genotype in all domestic pigs. All hybrids tested with the allele discrimination protocol revealed the presence of both alleles (CT). The sequenced wild boar sample represented the CC genotype and the pig sample represented the TT genotype.

## 4. Discussion

The *MC1R* polymorphism in wild boars and domestic pigs from Poland has previously been studied by [22] and [23]. Our study expands the number of wild boars tested in central Europe and enables comparison with findings in local domestic breeds. To our knowledge, this represents the first time that the *NR6A1* g.748C > T test has been implemented in the Polish population.

The efficacy of searching for markers suitable to determine these two subspecies relies on the type of genetic marker. Choosing markers on which strong selection has been conducted (e.g., *MC1R* and *NR6A1*) might be beneficial. *MC1R* is one of the pivotal genes that play a significant role in determining coat/skin colour. The *MC1R* gene polymorphism has already been proved useful in species identification of food products, e.g., authentication of Parmigiano Reggiano cheese produced only from Reggiana milk [24], or products containing meat from the Nero Siciliano pig [25]. However, basing a discriminatory analysis on only one marker in closely related taxa such as pigs and wild boars is inefficient [1,26]. Therefore, in this study, we also used the *NR6A1* SNP g.299084751 C > T (p.Pro192Leu). This marker is related to higher meat efficiency in pigs, due to the longer body achieved from more thoracic vertebrae [9]. Our results are consistent with studies conducted elsewhere [1,26]. Specifically, the homozygous genotype (TT) is prevalent in domestic breeds (Duroc, large white, and landrace pigs, Pulawska and Złotnicka White). In contrast, European wild boar demonstrated greater frequencies of the CC genotype. 

According to our results, none of the four diagnostic SNPs in *MC1R* could sufficiently differentiate the two subspecies. Nevertheless, we verified the unique *MC1R* profile of Duroc, classified as the red (e) allele [6]. This is consistent with the Duroc coat colour pattern, which is exceptional among other breeds studied (Figure S2). A unique profile has been classified under the most distant haplotype on the haplotype network. Unlike results obtained elsewhere [6,22,26], not all Durocs were e/e homozygotes in our study. Of particular interest is the wild type genotype (E^+^/E^+^) identified in one Duroc sample. STRUCTURE software analysis reflected these results and assigned the E^+^/E^+^ Duroc samples to the wild boar cluster. The identification of wild type alleles in commercial pig breeds is not common and was not found in the studies conducted by [8,26] and [22]. In Poland, Durocs have been used in breeding programmes to improve the meat efficacy of pig breeds (e.g., PL, PLW) [27]. Concerning the aim of the study, we consider that the presence of the wild allele in this breed might have in turn been transferred to other breeds, making it difficult to differentiate both subspecies. Further studies conducted on a larger number of samples should give an insight into the frequency of the E^+^/E^+^ genotype in domestic breeds. 

A specific e allele was found in two Złotnicka White samples. We have not found any mention in the literature of breeding schemes introducing Duroc breed into the Złotnicka White breed. Additionally, both Złotnicka samples with the recessive e allele were depicted as having mixed profiles, including Duroc and other domestic breeds. 

Admixture of the domestic pig genome with the wild boar population was identified in previous studies carried out on Polish [22,23] and European [26] population pigs and wild boars. Our study also found two wild boar samples representing hybrids of the wild type and domestic genotype (E^+^/^ED1,2,P2^) STRUCTURE just as haplotype files clustered these two wild boars accordingly. The identification of hybrids or genome admixture is not dependent on the marker(s) implemented. [28] studied microsatellites in domestic breeds, captive wild boars and free-living wild boars. They identified domestic pig gene introgression into the wild population due to keeping pigs in semi-enclosed farms without human supervision. A study of *MC1R* in Polish wild boars and domestic pigs also revealed high domestic pig gene introgression to wild boars, comparable to regions in which pigs are kept in semi-enclosed conditions [22]. The Fst value indicates a moderate genetic differentiation of both populations, however it is worth noting that Fst was calculated based on one type of marker mapped to loci under selection. A comprehensive insight into the genetic structure of a population and a better understanding of evolutionary processes requires the introduction of multiple loci or genome regions [29].

This first study of *NR6A1* g.748 C > T in Polish pigs and wild boars did not demonstrate hybrid profiles. This might affect strong selective pressure for body elongation to increase meat production and improve reproductive efficacy [9,30]. In the study conducted by [26], all domestic pig samples carried the mutated *NR6A1* allele, suggesting it was fixed in the population. Interestingly, in the same study, the domestic allele was found in 7.46% of European wild boars. As this is a preliminary study, such discrepancies should be clarified in a further study conducted on a larger number of samples. This could improve the power of both differentiating tests, which are conducted based on four polymorphic sites. 

## 5. Conclusions

None of the four diagnostic SNPs in *MC1R* could sufficiently differentiate the two subspecies. This goal was achieved through the analysis of *NR6A1* polymorphism. However, the *NR6A1* study did not reveal admixture profiles that were identified by the *MC1R* study. Therefore, we believe that discrimination of both species should be performed using polymorphic sites in both genes. 

## Figures and Tables

**Table 1:** Primer sequences used for amplification of *MC1R* and *NR6A1* regions, Taqman probes used for real-time PCR, and restriction enzymes used for *MC1R* genotype identification.

Gene	Primer	Reference	Sequence Length (nt)	SNP Position *	RFLP/Real-Time PCR	Alleles (Band Length in bp)
*MC1R*	F: AGTGCCTGGAGGTGTCCATTCAC R: CGTAGATGAGGGGGTCCAGGATAGA	[8]	575	370	BspHI	G (672), A (472, 200)
				491	-	CC, CT, TT
				727	BstUI	G (289, 222, 110), A (332, 289),
				729 **	-	A, G
*NR6A1*	F:AGGGCTTCAGAGAGCAACCA R:TGAAGCTCACCTGGAGGACAGT	[2]	18	748	VIC-TCACcGGGCTCCA-MGB NFQ FAM-CTCACtGGGCTCC-MGB NFQ	CC, CT, TT

F = forward primer, R = reverse primer; nt—nucleotide, bp—basepair; * SNP positions according to reference sequences No. NM_001008690 (*MC1R*) and AB248749 (*NR6A1*); ** based on sequence alignment analysis, this SNP is not polymorphic in samples studied.

**Table 2:** *MC1R* alleles identified within sequence alignment for the wild boars and domestic pigs.

	*MC1R Locus* */Genotype ** and No. of Animals									*MC1R* Genotypes *** and No. of Animals						
	g.370G > A			g.491C > T			g.727G > A									
Breed	GG	AG	AA	CC	CT	TT	GG	AG	AA	E^+^/E^+^	E^+^/E^D1,2,P2,3^	e/e	e/E ^D1,2,P2,3^	e/E^P3^	E^D1,2,P2^/, E^D1,2,P2,3^	ED^D1,2,P2^/E^P3^
WB (*n* = 24)	22	2	-	24	-	-	24	-	-	22	2	-	-	-	-	-
D (*n* = 17)	15	1	1	2	1	14	2	1	14	1		14	1		1	-
ZW (*n* = 13)	-	2	11	11	2	-	11	2	-	-		-	1	1	11	-
P (*n* = 10)	-	-	10	10	-	-	10	-	-	-	-	-	-	-	10	-
PL (*n* = 14)	-	-	14	14	-	-	14	-	-	-	-	-			4	10
PLW (*n* = 13)	-	-	13	13	-	-	13	-	-	-	-	-			13	

WB—Polish wild boar, D—Duroc, ZW—Złotnicka White, P—Puławska, PL—Polish Landrace, PLW—Polish Large White; * SNP positions according to reference sequence No. NM_001008690 (*MC1R*); ** identified in breeds studied; *** based on allele sequences available on GeneBank: E^+^ (AF082490, [6]), E^D1^ (AF082489, [6]); e (EU443691, [7]), E^P2^ (EU443722, [7]), E^D2^ (EU443685, [7]), E^P3^ (EU443726, [7]).

## Supplementary Materials

The following are available online at https://www.mdpi.com/article/10.3390/ani11072123/s1. Table S1. Contains the specific thermal condition of PCRs conducted in the study. Figure S1. Contains locations of samples studied. Figure S2. Contains pictures of pigs breeds studied. Figure S3. Contains haplotype network generated with NETWORK 4.6.1.6; Supplementary Material 1. Contains haplotype files.
