# Supplementary material for: Differentiating Pigs from Wild Boars Based on NR6A1 and MC1R Gene Polymorphisms
Source: Animals (Basel). 2021 Jul 17;11(7):2123. doi: 10.3390/ani11072123 (PMC8300376; doi:10.3390/ani11072123)
Supplement: Supplementary file 1 [file animals-11-02123-s001.zip › Supplementary Figure S2.pdf]

Supplementary Figure 2. Pictures of the pig breeds studied.

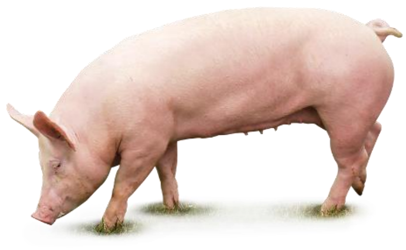

Polish Large White;
https://www.polsus.pl/index.php/en/pig-breeding/breeds/polish-large-white

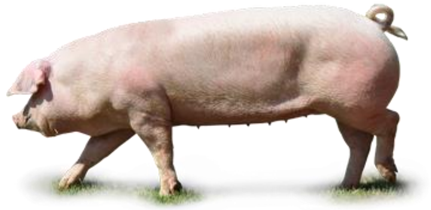

Polish landrace;
https://www.polsus.pl/index.php/en/pig-breeding/breeds/polish-landrace

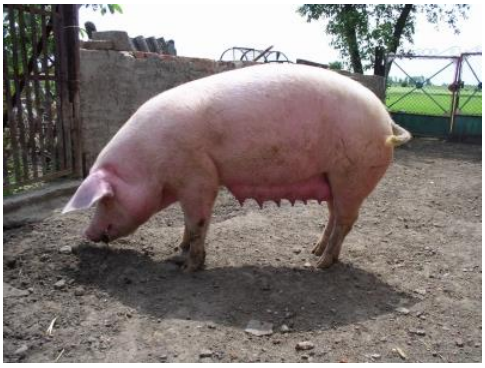

Złotnicka White;
http://www.bioroznorodnosc.izoo.krakow.pl/swinie/biala

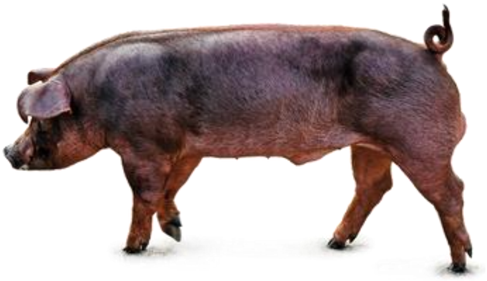

Duroc;
https://www.polsus.pl/index.php/en/pig-breeding/breeds/duroc

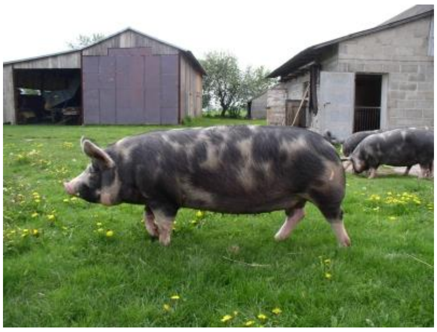

Puławska;
http://www.bioroznorodnosc.izoo.krakow.pl/swinie/pulawska
