# Supplementary material for: Differentiating Pigs from Wild Boars Based on NR6A1 and MC1R Gene Polymorphisms
Source: Animals (Basel). 2021 Jul 17;11(7):2123. doi: 10.3390/ani11072123 (PMC8300376; doi:10.3390/ani11072123)
Supplement: Supplementary file 1 [file animals-11-02123-s001.zip › Suppl_Table S1.pdf]

Table 1. Thermal condition of PCR used in the study

MC1R PCR				MC1R sequencing PCR			NR6A1 real-time PCR		
Reaction step	Temp.	time	cycles	temp	time	cycles	temp	time	cycles
Denaturation	95 ºC	15 min		95	10 min		95ºC	15 min.	1x
Annealing	94 ºC	45 sec	35	96	10 sec	25	95ºC	15 sec	45x
	63 ºC	50sec		55	4 min		60ºC	1 min.	
