# Supplementary figures and images for: Differentiating Pigs from Wild Boars Based on NR6A1 and MC1R Gene Polymorphisms
Source: Animals (Basel). 2021 Jul 17;11(7):2123. doi: 10.3390/ani11072123 (PMC8300376; doi:10.3390/ani11072123)

Figure S1. Sampling locations.

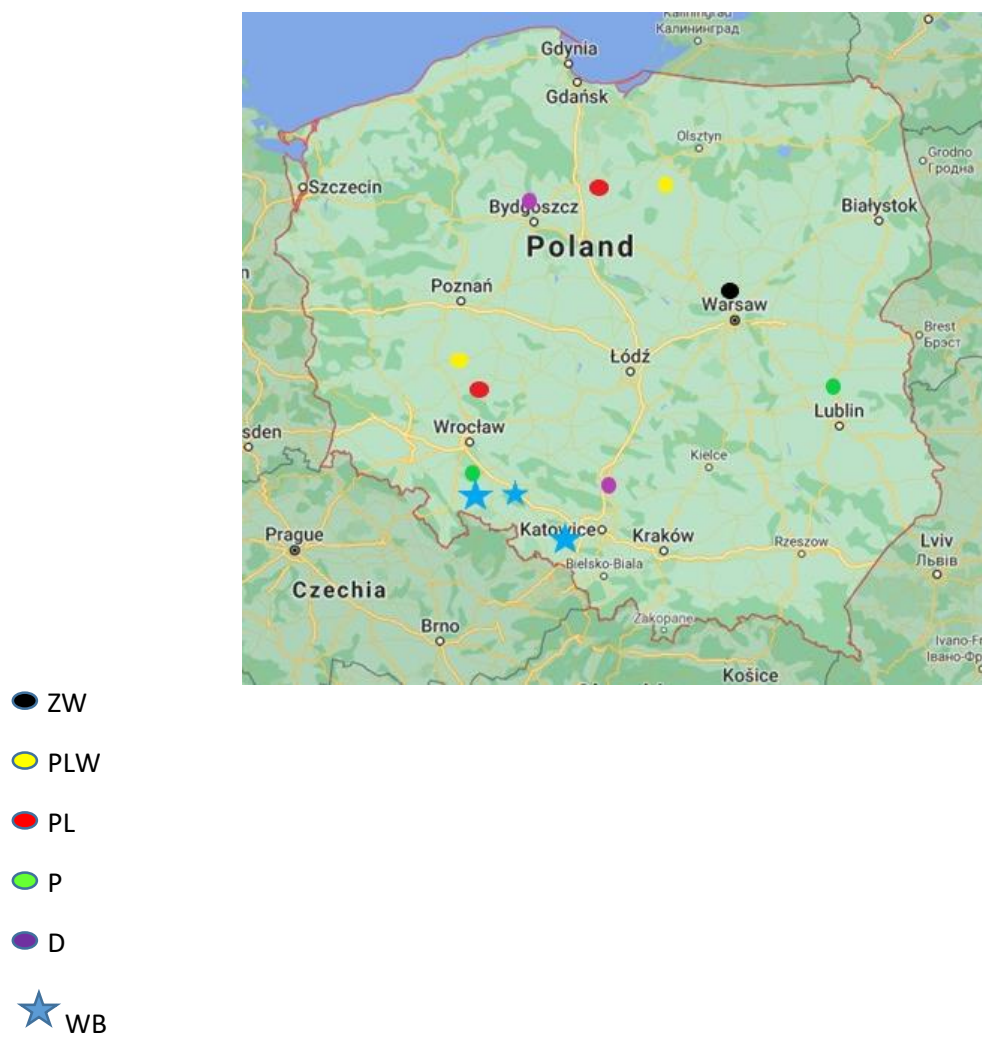

Supplement: Supplementary file 1 [file animals-11-02123-s001.zip › Supplementary Figure S1.pdf]

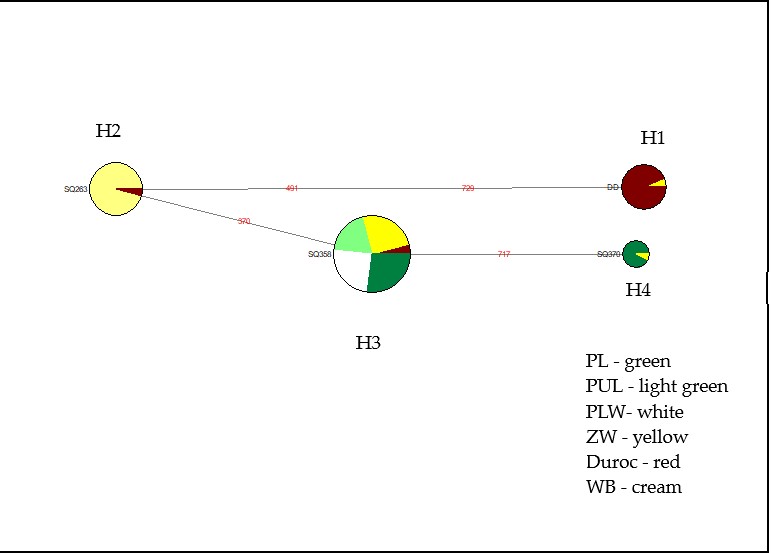

Supplement: Supplementary file 1 [file animals-11-02123-s001.zip › Supplementary Figure S3.jpg]
